# Selecting Representative Tumour Bed Slices May Allow Reduced Embedding Without Loss of Accuracy in Response Evaluation to Neoadjuvant Immunotherapy in Stage IIIB/C Cutaneous Melanoma

**DOI:** 10.3390/cancers18091400

**Published:** 2026-04-28

**Authors:** Anders Bergström, Axel Nelson, Anne Huibers, Emel Cicek, Åse Silverdal, Martin E. Johansson, Katarzyna Lundmark, Jonas Selling, Anders Muszta, Iva Johansson

**Affiliations:** 1 Västra Götalandsregionen, Department of Clinical Pathology, Sahlgrenska University Hospital, SE41335 Gothenburg, Sweden; anders.bergstrom@gu.se (A.B.); ase.silverdal@vgregion.se (Å.S.); martin.e.johansson@gu.se (M.E.J.); 2 Institute of Biomedicine, Sahlgrenska Academy, University of Gothenburg, P.O. Box 400, SE40530 Gothenburg, Sweden; 3 Västra Götalandsregionen, Department of Oncology, Sahlgrenska University Hospital, SE41335 Gothenburg, Sweden; axel.nelson@vgregion.se; 4 Institute of Clinical Sciences, Sahlgrenska Academy, University of Gothenburg, P.O. Box 400, SE40530 Gothenburg, Sweden; anne.huibers@vgregion.se; 5 Västra Götalandsregionen, Department of Surgery, Sahlgrenska University Hospital, SE41335 Gothenburg, Sweden; 6 Västra Götalandsregionen, Department of Clinical Pathology, Södra Älvsborgs Sjukhus, SE50182 Borås, Sweden; emel.karacan@vgregion.se; 7 Department of Clinical Pathology, Linköping University Hospital, SE58185 Linköping, Sweden; katarzyna.lundmark@regionostergotland.se; 8 Department of Biomedical and Clinical Sciences, Linköping University, SE58183 Linköping, Sweden; 9 Statistikakademin AB, SE75321 Uppsala, Sweden; jonas.selling@statistikakademin.se; 10 Sahlgrenska Academy and School of Public Health and Community Medicine, Institute of Medicine, University of Gothenburg, P.O. Box 400, SE40530 Gothenburg, Sweden; anders.muszta@gu.se

**Keywords:** melanoma, neoadjuvant immunotherapy, pathological response, tumour bed, residual viable tumour

## Abstract

**Background/Objectives:** Histological assessment of the response to neoadjuvant immunotherapy in stage IIIB/C cutaneous melanoma is commonly performed using the INMC recommendations, which advocate embedding the entire tumour bed. However, this approach is highly resource-intensive. The original Swedish National Clinical Cancer Care Guidelines for Melanoma recommend a reduced embedding strategy, but its diagnostic performance in comparison with full embedding has not been systematically evaluated. We aimed to assess whether a reduced sampling approach based on selecting representative tumour bed slices provides comparable response classification to comprehensive embedding according to the INMC protocol. **Methods:** Ten consecutive patients with stage IIIB/C melanoma treated with neoadjuvant immunotherapy and subsequent lymph node dissection were included. All lymph node material was completely embedded, and the pathological response was evaluated using INMC criteria. Response classification based on the full embedding of the tumour bed was compared with reduced-sampling approaches that simulate the Swedish National Clinical Cancer Care Guidelines for Melanoma. Agreement between sampling methods was analyzed. **Results:** Targeted reduced sampling of two slices per lymph node demonstrated complete agreement with the full embedding for the INMC response category. **Conclusions:** In our study, a targeted reduced-embedding strategy focusing on slices with the largest area of tumour bed enables accurate histological assessment of response to neoadjuvant immunotherapy in stage IIIB/C melanoma while substantially reducing workload. These findings support the feasibility of targeted reduced embedding of instructions in the Swedish National Clinical Cancer Care Guidelines for Melanoma as an efficient alternative to full embedding of the lymphadenectomy specimens.

## Simple Summary

Histological evaluation of the response to neoadjuvant immunotherapy in stage IIIB/C melanoma requires extensive tissue embedding in accordance with international protocols. Excessive embedding requirements increase the workload for pathology laboratories and may potentially impact patient safety by contributing to case backlogs and displacing other diagnostically urgent specimens. In this study, we examined the performance of reduced embedding instructions in the original Swedish National Clinical Cancer Care Guidelines for Melanoma compared with embedding all tumour bed tissue. We analyzed ten consecutive patients to determine whether selecting two to four tissue slices, either random or containing the largest tumour bed area, provided comparable response classification. The reduced targeted approach showed complete agreement with the International Neoadjuvant Melanoma Consortium (INMC) response category in all cases. Our findings suggest that the tissue slices with the largest tumour bed area contain the key information needed for response assessment, indicating that examining every slice may not be necessary.

## 1. Introduction

Neoadjuvant immune checkpoint blockade has emerged as an increasingly established treatment strategy for patients with resectable stage IIIB/C cutaneous melanoma. Combination therapy with anti-Cytotoxic T-lymphocyte-Associated Antigen (CTLA-4) and anti-Programmed Cell Death Protein 1 (PD-1), as well as anti-PD-1 monotherapy administered before surgery, has demonstrated high pathological response rates and signaled improved recurrence-free survival, particularly among patients who achieve a major pathological response [1,2,3,4,5,6,7,8,9,10]. An accumulating body of data from randomized trials and pooled analyses (OpACIN-neo, NADINA, PRADO, and SWOG S1801) has suggested improved survival benefits relative to adjuvant strategies. Even though the practice varies between institutions and guidelines, in many centers, the neoadjuvant approach is increasingly replacing the traditional adjuvant treatment strategies for high-risk stage III melanoma [1,2,11,12,13,14,15,16,17,18,19]. A central component of this treatment paradigm is the histopathological assessment of tumor response in resected lymph node specimens, which serves as a biomarker and guides subsequent therapeutic decision-making [17,20,21,22].

To standardize response assessment, the INMC introduced consensus guidelines recommending estimation of the percentage of residual viable tumour (%RVT) within the tumor bed. Accurate %RVT assessment according to the INMC protocol requires an extensive evaluation of the entire tumor bed [20,23]. For large specimens, this method generates a high number of tissue blocks, substantially increasing the workload for pathology departments and associated healthcare costs. This also poses a risk of potential displacement effects, which may disrupt routine diagnostic workflows and ultimately compromise patient safety [24,25,26]. The increase in pathology workload has been witnessed during the authors’ long experience in routine pathology. The extent of pathological sampling required for accurate assessment of the response to neoadjuvant therapy in melanoma remains a subject of active debate. While a comprehensive evaluation of the entire tumour bed may intuitively reduce the risk of missing residual disease, this assumption has not been definitively validated across all clinical contexts and may not be universally necessary. Emerging evidence from neoadjuvant trials suggests that more focused sampling strategies, such as evaluation of the index lymph node may provide clinically meaningful prognostic information and guide surgical de-escalation [27]. A recent study by Rawson et al. demonstrated that reducing the number of tumour-bed slices examined does not significantly compromise the accuracy of pathological response assessment, with %RVT and response classification remaining highly reliable after neoadjuvant immunotherapy in stage IIIB/C melanoma [28].

The INMC protocol and the Swedish National Guidelines represent two distinct strategies for pathological sampling in the neoadjuvant setting. The INMC approach is largely driven by the aim of maximizing diagnostic accuracy and minimizing the risk of overlooking residual disease. In contrast, the Swedish Guidelines adopt a more pragmatic perspective, taking into account real-world constraints such as sustainability and resource allocation within pathology services. Both the extensive INMC protocol and the recommendations outlined in the Swedish National Clinical Cancer Care Guidelines for Melanoma [29] are largely empirically driven and were developed to promote standardized and reproducible assessment of pathological response after neoadjuvant immunotherapy. The Swedish National Clinical Cancer Care Guidelines for melanoma, specifically regarding the handling of metastasis and lymphadenectomy specimens, approved in September 2023 and updated in June 2025, implement a reduced sampling strategy from the outset, in contrast to the INMC protocol, which recommends comprehensive evaluation of the entire tumour bed. However, the diagnostic performance of this reduced embedding strategy has not been systematically validated.

The accurate assessment of %RVT depends on a meticulous histopathological assessment of the tumour bed. The tumour bed encompasses not only areas containing viable melanoma cells but also therapy-related regression changes, such as acute and chronic inflammation, fibrosis, and necrosis. Accordingly, the INMC guidelines recommend complete submission of all macroscopically positive lymph nodes ≤ 3 cm and serial cross-sectional slices at 1 cm intervals for nodes > 3 cm, resulting in a potentially large number of tissue blocks, in some cases exceeding 100. The rationale for comprehensive sampling is to ensure consistency across melanoma neoadjuvant trials and to minimize the risk of missing small residual tumour deposits. In contrast, the Swedish Clinical Cancer Care Guidelines for Melanoma recommend embedding at least two slices per macroscopically positive lymph node ≤ 5 cm and at least four slices for nodes > 5 cm, containing the most representative tumour-bed areas. Given the current resource constraints in many pathology departments, it is plausible that the minimum recommended number of tissue slices from each tumour bed may often be submitted in routine practice.

Accordingly, there is a clear need to determine whether reduced examination of the tumour bed can provide response classification comparable to that obtained with more extensive protocols. In this single-center prospective study, conducted in a Swedish clinical context, we performed two simulation approaches to model reduced embedding: random selection and targeted selection of tissue slices containing the largest tumour bed area. We aimed to investigate how reduced sampling, random or targeted, performs compared to the INMC protocol.

## 2. Materials and Methods

### 2.1. Patients

We included ten consecutive patients with stage IIIB/C cutaneous melanoma treated with neoadjuvant immunotherapy followed by lymphadenectomy at Sahlgrenska University Hospital, Gothenburg, Sweden, between 17 March and 27 May 2025. The study was approved by the ethics review board (2023-08198-01).

### 2.2. Specimen Handling and Histopathological Evaluation

**Figure 1:**
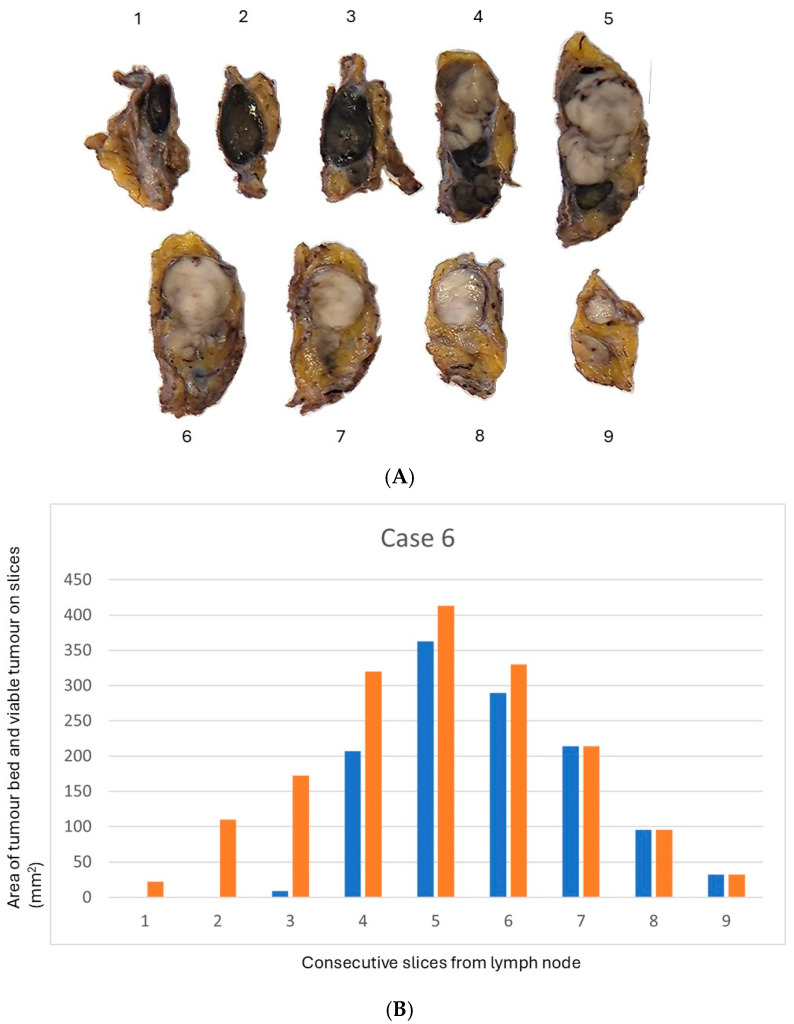
(**A**) Case 6. Macroscopic photograph of lymph node 1 cut into 9 slices. The slices with the largest tumour bed area were slices 5, 6, 4, and 7 in descending order. (**B**) Case 6. Histogram of lymph node 1 cut into 9 slices, showing the area of viable tumour (blue) within each slice (orange) measured on digitized whole slide images. Area of tumour bed and viable tumour on the y-axis. The consecutive slices numbered 1–9 on the x-axis (same number shown in (**A**)).

Formalin-fixed lymphadenectomy specimens from the axillary, iliacal, and inguinal regions were grossly dissected, and all lymph nodes were sectioned at 4 mm intervals (Figure 1A,B). Representative slices were embedded from all macroscopically tumour-free lymph nodes. In the next step, slices from lymph nodes with macroscopically identifiable tumour beds were entirely embedded and processed according to standard histopathological protocols (Figure 1A). Most specimens were processed in System III slotted tissue cassettes (CellPath Ltd., Newtown, UK; 30 × 25 × 5 mm). For large specimens, Tissue-Tec Super Mega-Cassettes (Sakura Finetec, Tokyo, Japan; 65 × 50 × 15 mm) were used. Paraffin sections (3 µm) were whole slide scanned in ×40 magnification (scanners Hamamatsu NanoZoomer S360 and S60, Hamamatsu Photonics, Hamamatsu City, Japan) and evaluated both digitally and using a brightfield microscope (Olympus BX53, Olympus, Tokyo, Japan) by two experienced senior dermatopathologists (IJ and ÅS). Tumour bed area and residual viable tumour were measured on digitized whole-slide images with manual annotation using Visiopharm software (version 2023.01; Visiopharm A/S, Hørsholm, Denmark). %RVT and pathologic response (pR) were assessed in accordance with the INMC protocol, using all histological sections from all lymph nodes containing tumour bed as the basis for the final case-level evaluation. Pathological response is categorized based on the proportion of viable melanoma within the tumour bed. Pathological complete response (pCR) is defined by the absence of viable tumour cells, whereas near complete response (pNCR) denotes the presence of viable melanoma involving a maximum of 10% of the tumour bed. pCR and pNCR are grouped together and referred to as major pathological response (MPR). Pathological partial response (pPR) is assigned when viable tumour comprises more than 10% but a maximum of 50% of the tumour bed, and pathological non-response (pNR) when viable melanoma exceeds 50% [23].

### 2.3. Statistical Analysis

#### 2.3.1. Mathematical Analysis of Outcomes Using Limited Random Sampling of 2–4 Slices

To assess the impact of limited sampling on the probability of correct classification, mathematical calculations of all possible scenarios resulting from limited sampling were performed on a per-case basis by an independent statistician. For each case, all available tumour bed-containing slices were considered, and all possible random combinations of two, three, and four slices from each lymph node containing a tumour bed were generated without replacement. For each random combination, the %RVT and corresponding response category were determined and compared with the reference classification based on the evaluation of all available slices from the same case. The proportion of correctly classified cases was calculated for each sampling level (2, 3, and 4 slices). The relationship between the number of examined slices and the probability of correct classification was derived from an exhaustive combinatorial simulation, in which all possible combinations of 2, 3, or 4 slices from each tumour bed were evaluated, and summarized using a nonlinear regression model derived from the sampling scenarios. This approach was chosen because the increase in classification accuracy is not expected to be linear; rather, it is expected to rise rapidly with additional slices and then gradually plateau as it approaches 100%.

#### 2.3.2. Simulation of Targeted Reduced Sampling of Slices of the Largest Tumour Bed

To simulate a targeted sampling strategy, slices were selected based on the largest tumour bed area (Figure 1). For each case, all tumour bed-containing slices were reviewed, and slices with the largest tumour bed area were identified. The %RVT and corresponding response category were then determined for each case using only the selected slices, simulating the possible scenarios with two, three, and four slices, respectively. The results from these targeted sampling scenarios were then compared with the reference classification obtained from the assessment of all available slices from the same case. The accuracy of classification was calculated on a per-case basis for each sampling level (2, 3, and 4 slices with the largest tumour bed area).

The reference for assessing percentage residual viable tumor (%RVT) was the INMC method, and targeted reduced-sampling approaches (2, 3, and 4 slices) were compared with this reference. Data were paired because all methods were applied to the same patients (n = 10), and %RVT values were skewed with a floor effect; therefore, nonparametric and agreement-based methods were selected.

The primary endpoint was agreement in pathological response classification, defined as pCR (0%RVT), pNCR (≤10%RVT), pPR (>10%RVT ≤ 50), and pNR (>50%RVT). These response categories were treated as ordinal variables. Agreement between each reduced sampling method and the gold standard (INMC) was assessed using quadratic weighted Cohen’s kappa to account for the ordinal structure of the categories and to appropriately penalize larger misclassifications. Cross-tabulation analyses were additionally performed to calculate percentage concordance with the gold standard.

Secondary analyses evaluated agreement in continuous %RVT between reduced sampling methods and INMC. Agreement was assessed using classic Bland–Altman analysis, showing the mean difference (bias) with 95% confidence intervals and limits of agreement (LoA) composed of ±1.96 standard deviations of the bias and its 95% confidence intervals. In addition, intraclass correlation coefficients (ICC) were calculated using a two-way mixed-effects model with absolute agreement and single measures to quantify the strength of agreement between each reduced sampling method and the gold standard.

Continuous variables were summarized using medians and ranges due to non-normal distribution and small sample size, while categorical variables were reported as frequencies and percentages. A two-sided *p*-value < 0.05 was considered statistically significant.

Statistical analyses were performed using IBM SPSS Statistics Version 30.0.0.0 (172) (IBM Corp., Armonk, NY, USA).

## 3. Results

### 3.1. Study Cohort

A total of 10 consecutive patients with stage IIIB/C melanoma were included (Table 1). The median age was 62 years (min-max 49–79 years) and 40% were female. The median time from excision of the primary melanoma to recurrence was 2 years (min-max 0–13 years). BRAF V600E or V600K mutations were detected in 5 patients (50%). Patients received neoadjuvant PD1 inhibitor monotherapy: 9 of 10 received two cycles of nivolumab (6 mg/kg), and 1 of 10 received three cycles of pembrolizumab (200 mg). Therapeutic lymphadenectomy was performed at a median of 7.6 weeks (min-max 6.7–13.7 weeks) after treatment initiation.

### 3.2. Specimen Characteristics

The study material comprised lymphadenectomy specimens from the axillary (n = 6), iliacal (n = 2), and inguinal (n = 2) nodal basins. The median number of lymph nodes per case was 11 (min-max 2–21). Tumour bed was identified in all cases, involving a median number of 2 lymph nodes per case (min-max 1–5). 115 lymph nodes were submitted for histological examination, yielding 325 tissue slices embedded in 240 tissue blocks. 25 lymph nodes contained tumour bed, median lymph-node size was 30 mm (interquartile range 24–43 mm, min–max 8–195 mm). 191 blocks were processed using standard System III slotted cassettes (CellPath Ltd., UK; approximately 30 × 25 × 5 mm) and 49 were processed in Tissue-Tec Super Mega-Cassettes (Sakura Finetek, Nagano, Japan, approximately 65 × 50 × 15 mm).

### 3.3. Histopathological Findings

According to INMC criteria (Table 2), four of ten patients (40%) achieved complete response (pCR), two (20%) had near-complete response (pNCR), and four (40%) showed no response (pNR). The median %RVT was 2.9% (range, 0–92%), with four cases demonstrating 0% RVT and four cases exhibiting high residual tumour burden (>50% RVT). INMC classification, based on the evaluation of all tumour bed-containing slices, was used as the reference for subsequent sampling simulations.

### 3.4. Simulation of Random Sampling of 2–4 Slices

A random-sampling simulation demonstrated that the probability of correct per-case classification increased with the number of slices examined. When two randomly selected slices from each lymph node with a tumour bed were evaluated in every case, the probability of correct classification compared with the reference classification based on all slices was approximately 0.75. Analysis of three randomly selected slices increased the probability of correct classification to approximately 0.85, while evaluation of four randomly selected slices resulted in a probability of correct classification of approximately 0.94. The greatest improvement in classification accuracy was observed when increasing the number of examined slices from two to three, whereas additional gains beyond four slices were more modest.

### 3.5. Simulation of Targeted Sampling of 2–4 Slices of the Largest Tumour Bed

Simulation of targeted sampling using 2-, 3-, and 4 slices with the largest tumour bed area from each lymph node containing tumour bed showed perfect agreement in pathological response classification with the INMC reference (Table 3) (weighted κ = 1.00; 100% concordance). Continuous %RVT measurements demonstrated excellent agreement (ICC, two-way mixed, absolute agreement, single measures = 0.989 (95% CI: 0.955–0.997, *p* < 0.001). The Bland–Altman analysis showed a small positive or negative bias (d) depending on whether 2-, 3-, or 4 slices were used. For 2 slices = −0.235% (95% CI: −4.884 to 4.414), for 3 slices = 0.225% (95% CI: −2.832 to 3.282), and for 4 slices = −1.435% (95% CI: −4.400 to 1.530), which shows an acceptable agreement. The lower limits of agreement (LoA) for 2 slices were from −13.233 (95% CI: −21.578 to −4.888), and the upper LoA was 12,763 (95% CI: 4.418 to 21.109) (Table A2). No significant differences in %RVT were observed between any of the three targeted-sampling strategies and full assessment (Wilcoxon signed rank test, z = −0.405, *p* = 0.686, r = −0.181 for 2 slices) (Table A1).

## 4. Discussion

In this study, we demonstrate that the targeted reduced-embedding strategy provides histopathological response assessment comparable to the comprehensive INMC protocol. Targeted reduced sampling of the tissue slices with the largest tumour bed area yielded identical pathological response classification. Accurate categorization is essential for appropriate patient management. Although the proportion of tumour bed area assessed decreased with reduced sampling (median 84%, range (40–98%) for 4 slices; median 69%, range (31–93%) for 3 slices, and median 50.5%, range (21–88%) for 2 slices), the results remained highly consistent with INMC %RVT. The reduced tumour-bed coverage (as low as 21%) may risk missing spatial heterogeneity despite the consistency with full coverage in this study. The strong statistical agreement with the INMC protocol, including perfect concordance in response classification, excellent ICC for %RVT, minimal bias in the Bland–Altman analysis, and no significant differences between reduced and full sampling, suggests that limited targeted sampling may be sufficient in this context, although external validation is still needed.

These findings indicate that a pragmatic, resource-efficient embedding strategy can be implemented without compromising diagnostic accuracy in stage IIIB/C melanoma treated with neoadjuvant immunotherapy. The robustness of classification despite reduced sampling suggests that tumour bed slices with the largest area capture the biologically relevant distribution of residual viable tumour.

Targeted embedding of the largest tumour bed slices is likely to provide a more representative estimate of global %RVT than random sampling of slices of varying sizes (Figure 1B). As %RVT is an area-based metric, larger slices with tumour bed contribute proportionally more to the overall tumour bed assessment and better reflect the spatial heterogeneity of regression and residual tumour. In contrast, small slices may disproportionately sample focal tumour deposits or regressive areas, increasing variability and the risk of over- or underestimation. Thus, area-informed reduced sampling represents a biologically and quantitatively robust alternative to random reduced sampling. Importantly, the selection of slices for embedding needs to be based solely on the macroscopic tumour bed area rather than the macroscopic appearance of its contents, to avoid selection bias with enrichment of either regression-dominated areas or foci of viable tumour. All available slices from each lymph node need to be examined regarding the presence of the tumour bed, and the slices with the largest tumour bed are selected. The size of the tumour bed should be measured during grossing if multiple slices demonstrate tumour beds of similar sizes. In a typical case, the tumour bed is readily distinguished from normal tissue by an experienced pathologist.

Continuous %RVT measurements also showed good agreement with the INMC results, supported by a very high ICC and small bias. The small bias observed with reduced sampling was minimal and unlikely to be clinically meaningful given the categorical thresholds used in the INMC framework (Table A2). Although the Bland–Altman analysis showed relatively wide limits of agreement for some targeted sampling strategies, these discrepancies did not cross clinically relevant thresholds in our cohort, as the pathological response category remained unchanged in all cases. The clinically relevant cutoffs of 10% and 50% RVT define the boundaries between major pathological response, partial response, and no pathological response, respectively. Cases with %RVT values near category cut-offs may be more vulnerable to misclassification. Based on these findings, we propose that initial sampling should include two sections from each lymph node containing a tumour bed. If the global percentage of RVT falls within ±5% of clinically relevant cutoffs (10% or 50%), additional slices should be embedded to minimize the risk of misclassification of pathological response. In our study, only one case presented with a lymph node larger than 5 cm; however, this node also demonstrated complete concordance with the INMC results when assessed using two slices. This finding may suggest that the lymph node diameter criterion in the Swedish Cancer Care Guidelines could potentially be reconsidered or omitted.

**Figure A1:**
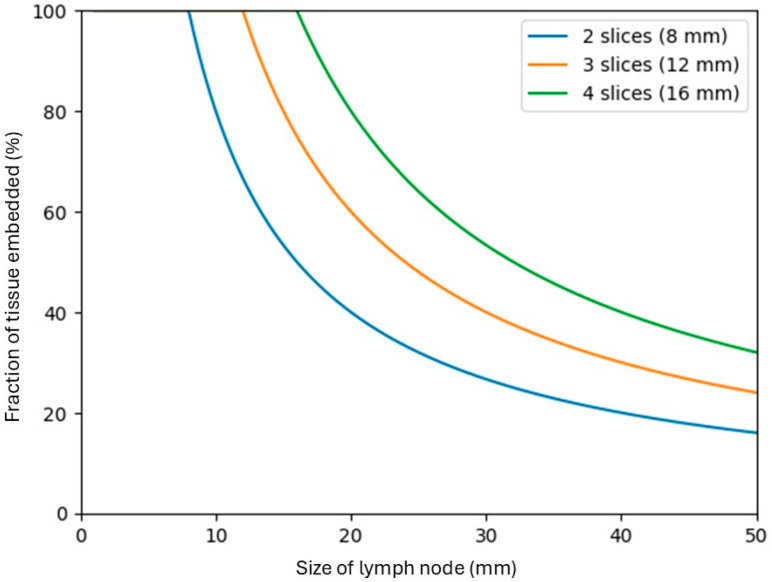
Relationship between lymph node size and the proportion of tissue embedded, stratified by the number of tissue slices processed. The x-axis represents lymph node size (mm), and the y-axis shows the percentage of total lymph node tissue embedded. Curves illustrate scenarios with 2 slices (8 mm total thickness), 3 slices (12 mm), and 4 slices (16 mm). For a given number of slices, the percentage of tissue embedded decreases with increasing lymph node size.

Only a limited number of studies have explored alternative sampling strategies for pathological response evaluation in melanoma or other malignant tumours. Weissferdt et al. performed a simulation analysis of 31 pretreated lung cancer resections comparing complete tumour bed submission with traditional sampling (1 slice per cm) [30]. They demonstrated that conventional sampling of neodjuvant-treated non-small-cell lung carcinoma frequently results in suboptimal accuracy for estimating %RVT and determining major or complete pathological response. Their results suggest that submitting up to a maximum of 20 slices provides the most accurate %RVT, MPR, and pCR assessment while remaining a practical approach for routine pathology practice. Our findings are consistent with Rawson et al., who demonstrated that reduced tumour bed assessment does not compromise the accuracy of %RVT evaluation or pathological response classification. Their study included 134 patients with stage IIIB/C melanoma treated with neoadjuvant immunotherapy. In part of their cohort, pathological assessment was limited to the index lymph node, and completion lymph node dissection was omitted in patients with a major pathological response. In contrast, our analysis was based on all lymph nodes, and all slices containing the tumour bed were evaluated, providing a comprehensive assessment of treatment response. Furthermore, whereas Rawson et al. focused on proportional reduction in embedded slices, our study modelled a targeted, area-based sampling strategy that more closely reflects real-world embedding decisions. The excellent agreement observed in our cohort, despite a substantial reduction in sampled tissue, suggests that the representativeness of the largest tumour bed area is more critical than the absolute number of slices examined (Table 3 and Figure A1 in Appendix A).

These findings support the Swedish Cancer Care Guidelines for Melanoma-reduced-embedding approach as a pragmatic and safe strategy that reduces workload while preserving diagnostic fidelity through targeted sampling of at least two slices per each macroscopically positive lymph node ≤ 5 cm and at least four slices for each lymph node > 5 cm with the most representative tumour bed area. Handling of specimens for response-evaluation after neoadjuvant therapy requires a high degree of experience, collaboration with the referring oncologic surgeon, and correlation to clinical and radiological findings. In our real-world study, referring clinicians always have access to radiological imaging, including MRI, and provide relevant clinical and radiological information in the pathology referral. Surgical specimens are always routinely carefully dissected in toto, and all lymph nodes—both tumor-free and those containing tumor bed—are identified and processed. To assess pathological response to neoadjuvant therapy, the evaluation subsequently focuses on lymph nodes containing a clearly identifiable tumor bed, as described in this study.

### 4.1. Limitations and Strengths of the Study

**Figure A2:**
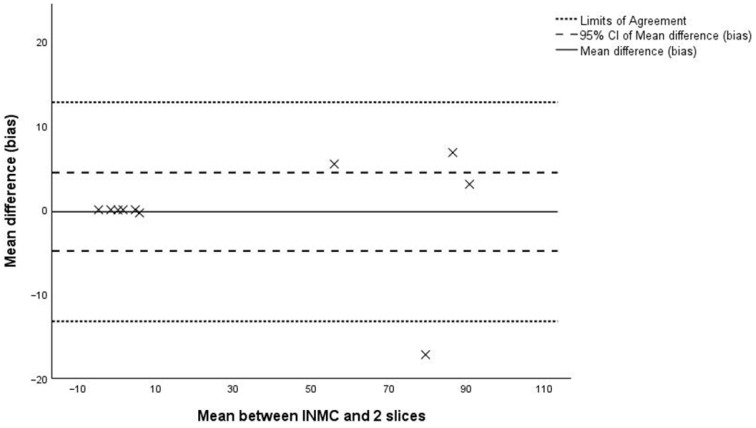
Bland- Altman plot comparing methods INMC and 2 slices. Solid line shows the mean difference (bias) with its associated 95% confidence interval (dashed line). Dotted lines show the upper and lower limits of agreement.

This study has several limitations. The sample size was small (n = 10) and was derived from a single centre, which may limit generalizability and statistical power. The short inclusion period may also introduce selection bias. The number of lymph nodes per case varied (range 2–21), reflecting real-world clinical practice but introducing potential sampling heterogeneity. Additionally, in our prospective consecutive cohort, there were no cases with pPR, which may also limit generalizability. Especially, the Bland–Altman analysis suffers from this, and it shows in a wide LoA. The Bland–Altman analysis also shows greater variation in pNR cases, which might suggest that observations with results close to 50%RVT should be examined with extra care (Figure A2). However, the paired within-case design, full tumour bed assessment, and concordant results across multiple agreement metrics (perfect weighted kappa and excellent ICC) strengthen the internal validity of the findings. In addition, the study was limited to lymph node specimens from stage IIIB/C melanoma, and extrapolation to other tumour types or anatomical sites requires further investigation. One patient received a different treatment (pembrolizumab vs. nivolumab); however, both the pathological response and the simulation results remained consistent with the overall findings.

Despite these limitations, the study has several strengths. The prospective, real-world design, full tumour bed assessment provides a robust internal reference standard. Agreement between full and targeted reduced sampling was consistently high across multiple complementary metrics, including perfect concordance in pathological response classification (weighted κ = 1.00), excellent reliability for continuous %RVT (ICC 0.989, 95% CI: 0.955–0.997), minimal bias in Bland–Altman analyses (−1.435% to 0.225%), and small to moderate effect sizes (r ranging from −0.099 to 0.431). Despite the small cohort, this study contributes to the limited body of literature on alternative sampling strategies and is based on a nationally implemented strategy.

### 4.2. Future Directions

Future studies in larger cohorts are warranted to validate these findings. Prospective validation across varying specimen sizes and response patterns would be particularly valuable. Additionally, the pathologists’ approach to selecting tissue for embedding may influence the results and warrants further investigation.

## 5. Conclusions

Our findings support the use of a reduced sampling protocol, as recommended in the current Swedish National Clinical Guidelines for neoadjuvant-treated melanoma lymphadenectomy specimens. In direct comparison with full embedding, targeted reduced sampling demonstrated complete concordance in pathological response classification, corresponding to 100% diagnostic accuracy in this cohort. We suggest refining by embedding slices with the largest tumor bed area, with 2 slices for each lymph node ≤ 5 cm and 4 slices for lymph nodes > 5 cm. For cases close to the pathological category boundaries (10% and 50%), additional sampling may improve accuracy. However, selecting slices based on tumour bed area may bias sampling towards dominant regions and risk missing intratumoural heterogeneity. Our results are based on a small cohort and warrant validation in larger cohorts with complete or near-complete embedding of the lymph node specimens.

## Figures and Tables

**Table 1:** Study Cohort.

Id	Age	Sex (M/F)	Nodal Basin	Primary Site	Primary Type	T Stage	Stage	BRAF	Neoadj Therapy
1	49	M	Iliacal	leg	NM	T4b	IIIC	wt	Nivo 6 mg/kg
2	78	M	Axilla	trunk	NM	T3a	IIIB	wt	Nivo 6 mg/kg
3	60	M	Axilla	trunk	NM	T3b	IIIC	V600E/K	Nivo 6 mg/kg
4	77	M	Axilla	trunk	SSM	T1a	IIIB	V600E/K	Nivo 6 mg/kg
5	52	F	Iliacal	leg	SSM	T1b	IIIB	V600E/K	Nivo 6 mg/kg
6	77	M	Inguinal	leg	NM	T3a	IIIC	wt	Nivo 6 mg/kg
7	62	F	Inguinal	leg	unknown	T2a	IIIC	unknown	Pembro 200 mg
8	63	F	Axilla	trunk	SSM	T2a	IIIC	V600E/K	Nivo 6 mg/kg
9	58	F	Axilla	arm	SSM	T2b	IIIB	V600E/K	Nivo 6 mg/kg
10	79	M	Axilla	arm	unknown	T3b	IIIC	wt	Nivo 6 mg/kg

Abbreviations: SSM superfial spreading melanoma, NM nodular melanoma, wt wild type.

**Table 2:** Results according to the INMC protocol.

Id	Lymph Nodes with Tumour Bed (n)/Total Number of Lymph Nodes (N) (n/N)	Number of Blocks with Tumour Bed (n)	Total Area of Tumour Bed (mm^2^)	Total Area of Viable Tumour (mm^2^)	%RVT According to INMC Protocol	Pathological Response Category INMC
1	5/5	22	1173	66	5, 6	pNCR
2	1/17	5	182	0	0	pCR
3	1/21	5	235	0	0	pCR
4	5/19	30	3893	0	0	pCR
5	2/9	7	1626	1460	90	pNR
6	1/6	12	1709	1210	71	pNR
7	2/2	8	735	2	0, 3	pNCR
8	4/8	13	430	0	0	pCR
9	2/13	15	3038	2802	92	pNR
10	2/15	51#	49,714	29,146	59	pNR

Abbreviations: %RVT percentage of residual viable tumour, INMC International Neoadjuvant Melanoma Consortium; #Slices from Case ID 10 were embedded in 49 large-size Tissue-Tec Super Mega-Cassettes and 2 standard-size cassettes.

**Table 3:** Results of targeted reduced embedding of 4, 3, and 2 slices with the largest tumour bed area per lymph node compared to the INMC protocol.

Id	% RVT				pR				Percentage of the Assessed Tumour Bed Area Compared to INMC Protocol (%)		
	INMC	4 Slices	3 Slices	2 Slices	INMC	4 Slices	3 Slices	2 Slices	4 Slices	3 Slices	2 Slices
1	5, 6	6, 5	4, 8	6	pNCR	pNCR	pNCR	pNCR	73	59	44
2	0	0	0	0	pCR	pCR	pCR	pCR	96	84	58
3	0	0	0	0	pCR	pCR	pCR	pCR	97	87	75
4	0	0	0	0	pCR	pCR	pCR	pCR	75	60	44
5	90	90	87	83	pNR	pNR	pNR	pNR	93	76	57
6	71	84	81	88	pNR	pNR	pNR	pNR	75	62	44
7	0, 3	0, 2	0, 2	0, 3	pNCR	pNCR	pNCR	pNCR	98	81	60
8	0	0	0	0	pCR	pCR	pCR	pCR	96	93	88
9	92	93	91	89	pNR	pNR	pNR	pNR	62	51	38
10	59	58	52	53	pNR	pNR	pNR	pNR	40	31	21

Abbreviations: %RVT percentage of residual viable tumour, pR pathological response, INMC International Neoadjuvant Melanoma Consortium, pNCR pathological near complete response, pCR pathological complete response, pNR pathological no response.

## Data Availability

Data supporting reported results are stored in anonymised form by the authors.

## Abbreviations

The following abbreviations are used in this manuscript:

INMC	International Neoadjuvant Melanoma Consortium
CTLA-4	Cytotoxic T-Lymphocyte-Associated Antigen 4
PD1	Programmed Cell Death Protein 1
%RVT	Percentage of residual viable tumour
pR	Pathological response category
pCR	Pathological complete response
pNCR	Pathological near complete response
MPR	Major pathological response
pPR	Pathological partial response
pNR	Pathological non-response
LoA	Limits of agreement
ICC	Intraclass correlation coefficient
NM	Nodular melanoma
SSM	Superficial spreading melanoma

## Appendix A

### Appendix A.2

**Table A1:** Comparison between full assessment and slice-based methods using non-parametric statistics.

Method	Z-Value	*p*-Value	Effect Size (r)
2 slices	−0.314	0.753	−0.099
3 slices	−0.943	0.345	−0.298
4 slices	1.363	0.173	0.431

Abbreviations: r, effect size; *p*-values calculated using the Wilcoxon signed-rank test.

### Appendix A.3

**Table A2:** Bland–Altman bias and 95% limits of agreement (LoA) with corresponding 95% confidence intervals for the different slice methods.

Method	Bias	95% CI	Lower LoA	95% CI	Upper LoA	95% CI
2 slices	−0.235	−4.884 to 4.414	−21.578	−13.233 to −4.888	12.763	4.418 to 21.109
3 slices	0.225	−2.832 to 3.282	−13.809	−8.322 to −2.834	8.772	3.284 to 14.259
4 slices	−1.435	−4.400 to 1.530	−15.046	−9.724 to −4.402	6.854	1.543 to 12.177

Abbreviations: CI, confidence interval; LoA, limits of agreement.
